# Patterns of Sociodemographic and Clinicopathologic Characteristics of Stages II and III Colorectal Cancer Patients by Age: Examining Potential Mechanisms of Young-Onset Disease

**DOI:** 10.1155/2017/4024580

**Published:** 2017-01-23

**Authors:** Caitlin C. Murphy, Hanna K. Sanoff, Karyn B. Stitzenberg, John A. Baron, Jennifer L. Lund, Robert S. Sandler

**Affiliations:** ^1^Division of Epidemiology, Department of Clinical Sciences, University of Texas Southwestern Medical Center, Dallas, TX, USA; ^2^Department of Epidemiology, The University of North Carolina at Chapel Hill, Chapel Hill, NC, USA; ^3^Lineberger Comprehensive Cancer Center, The University of North Carolina at Chapel Hill, Chapel Hill, NC, USA; ^4^Center for Gastrointestinal Biology and Disease, The University of North Carolina at Chapel Hill, Chapel Hill, NC, USA

## Abstract

*Background and Aims.* As a first step toward understanding the increasing incidence of colorectal cancer (CRC) in younger (age < 50) populations, we examined demographic, clinicopathologic, and socioeconomic characteristics and treatment receipt in a population-based sample of patients newly diagnosed with stages II and III CRC.* Methods.* Patients were sampled from the National Cancer Institute's Patterns of Care studies in 1990/91, 1995, 2000, 2005, and 2010 (*n* = 6, 862). Tumor characteristics and treatment data were obtained through medical record review and physician verification. We compared sociodemographic and clinicopathologic characteristics and treatment patterns of younger (age < 50) and older (age 50–69, age ≥ 70) CRC patients.* Results.* Younger patients were more likely to be black (13%) and Hispanic (15%) than patients aged 50–69 years (11% and 10%, resp.) and ≥70 years (7% each). A larger proportion of young white (41%) and Hispanic (33%) patients had rectal tumors, whereas tumors in the right colon were the most common in young black patients (39%). The majority of younger patients received chemotherapy and radiation therapy, although receipt of microsatellite instability testing was suboptimal (27%).* Conclusion.* Characteristics of patients diagnosed with young-onset CRC differ considerably by race/ethnicity, with a higher proportion of black and Hispanic patients diagnosed at the age of < 50 years.

## 1. Introduction

Incidence of colorectal cancer (CRC) in younger adults (age < 50 years) is rising in the US [1–3]. Despite an aging population, by 2030, approximately 11% of colon and 23% of rectal cancers are expected to be diagnosed in patients below the age of 50 [3]. Underlying mechanisms contributing to this increase are poorly understood, and reasons for the increase in CRC incidence in younger populations remain largely unknown.

Understanding differences in sociodemographic and clinicopathologic characteristics of CRC patients by age may provide an important insight into mechanisms that have contributed to increasing incidence of CRC in younger populations. However, most research in this area is limited to single institution settings or clinic-based samples. Findings from these studies generally reflect characteristics of patients treated at that institution. For example, a number of studies [4–6] show that proximal colon cancers are more common in younger patients, while others report a higher proportion of distal colon or rectal cancers [7–10] in this age group—or even no difference in anatomic subsite by age [11–16]. Relying on results from clinic-based samples may lead to inappropriate conclusions regarding the relative importance of sociodemographic and clinicopathologic characteristics in the development of young-onset CRC because there are differences in patient demographics (e.g., race/ethnicity, age) across institutions. As a consequence, we lack an understanding of the characteristics of and factors contributing to young-onset CRC in diverse settings and populations.

To address this gap in the literature, we examined demographic, socioeconomic, and clinicopathologic characteristics of younger and older CRC patients in a population-based sample of stages II and III CRC. We limited the study to stages II and III patients because we expected more variation in treatment with chemotherapy and radiation therapy. As a secondary aim, we also examined the receipt of treatment by age.

## 2. Methods

### 2.1. Study Population

The study population was derived from the National Cancer Institute's (NCI) Patterns of Care (POC) studies. The NCI annually conducts POC studies on a random sample of patients with select cancers (e.g., breast [17–19], colorectal [20–23], and cervical [24]) to complement data routinely collected through the Surveillance, Epidemiology, and End Results (SEER) program of cancer registries. Because chemotherapy administered in outpatient settings is often underascertained by SEER registries (i.e., SEER is primarily hospital-based), POC studies provide important information on the extent to which adjuvant therapies are delivered in community settings. Stages II and III CRC patients in participating SEER registries were included in POC studies in 1990, 1991, 1995, 2000, 2005, and 2010 [21]. Patients were stratified by registry, sex, age, and race/ethnicity, and a random sample was taken from within each stratum. There was oversampling by race/ethnicity in 1995, 2000, 2005, and 2010 to obtain more stable estimates. Patients were ineligible for POC studies if they were below the age of 20, previously diagnosed with cancer (excluding nonmelanoma skin cancer), diagnosed at autopsy or on death certificate only, or diagnosed with a synchronous cancer. For purposes of this analysis, we further excluded patients with tumors in the appendix (*n* = 4), who did not undergo cancer-directed surgery (*n* = 171), or with incomplete information to determine TNM staging (*n* = 18).

### 2.2. Covariates

We examined demographic, clinicopathologic, and socioeconomic characteristics of the study population. Patient demographics included age at diagnosis, sex, race/ethnicity (non-Hispanic white, non-Hispanic black, Hispanic, or others), and insurance (private, Medicare only, any Medicaid, or none).

Clinicopathologic features included tumor site, stage at diagnosis, histologic grade (well/moderately differentiated, poorly/undifferentiated), mucinous or signet ring cell histology, and receipt of microsatellite instability (MSI) testing. Tumor site included right colon (cecum, ascending colon, hepatic flexure, and transverse colon), left colon (splenic flexure, descending colon), sigmoid colon, and rectum (rectosigmoid junction, rectum) according to the International Classification of Disease for Oncology, 3rd Edition (ICD-O-3). Data on MSI were collected in 2010 only.

Socioeconomic indicators were derived from POC, SEER, and the Area Health Resource File (AHRF). POC contains patient-level data on hospital type (private, government, or nonprofit), an approved residency training program, total bed size, and cancer clinical trial enrollment. We also used a composite census-tract index of socioeconomic status based on measures developed by Yost et al. [25], including occupation, unemployment, poverty, education, income, and housing. The index was constructed to assess the relationship between socioeconomic status and cancer incidence using SEER data, as described elsewhere [26]. Data used in the index were derived from Census 2000 and American Community Survey 2005–2009 and reflect the populations and census tracts covered by the SEER 17 registries. The index (measured in quintiles) was available for study years 2000, 2005, and 2010. In addition, study data were linked with the AHRF, an extensive county-level database of socioeconomic indicators maintained by the U.S. Department of Health and Human Services [27]. We used AHRF data on per capita income, median household income, education level (% of persons aged ≥25 years with less than a high school diploma, high school or more, or four or more years of college), poverty (% of persons living below poverty line), unemployment (unemployment rate), total number of active physicians, and total number of gastroenterologists. Cutpoints for all AHRF variables were based on approximate tertiles. Income measures were adjusted to 2010 dollars.

Treatment patterns included type of surgery (partial, subtotal, or total colectomy or proctectomy), number of lymph nodes examined (0, 1–11, or ≥12), receipt of chemotherapy, and receipt of radiation therapy (among rectal cancer patients only). As part of POC studies, treatment information was abstracted from medical records and verified by treating physicians. Treating physicians were also asked to provide names and addresses of other physicians who may have treated the patient, who were subsequently contacted for additional treatment details.

### 2.3. Statistical Analysis

Descriptive statistics (e.g., proportions, means) were used to examine the distribution of covariates by age at diagnosis (<50 years, 50–69 years, and ≥70 years). Age categories were chosen in an effort to account for the potential heterogeneity of CRC in older patients (e.g., the CIMP phenotype is most common in female CRC patients aged ≥70 years [28]). Sensitivity analyses that considered different categorizations of age at diagnosis (e.g., <50 years, ≥50 years or <50 years, 50–64 years, or ≥65 years) did not appreciably change the results; therefore, we report the results of the primary analysis only. Comparisons between younger and older patients were performed using the Wald chi-square test and based on differences between observed and expected weighted frequencies [29].

To account for potential differences by race/ethnicity, we conducted a stratified analysis of select covariates in the subgroup of younger (age < 50 years) non-Hispanic white (*n* = 317), non-Hispanic black (*n* = 200), and Hispanic (*n* = 189) patients.

We also examined the proportion of patients who received chemotherapy and radiation therapy by tumor site (colon versus rectum), stage at diagnosis, and age. Patients who received therapy or patients for whom it was recommended that they receive therapy but it was unknown whether they did were considered to have received therapy (*n* = 91); patients who refused therapy (*n* = 221) were not considered to have received therapy.

Proportions and means were weighted with stratum-specific sample weights to reflect the population (i.e., SEER) from which the sample was drawn. Sample weights were calculated as the inverse of the sampling proportion for each sampling stratum.

All statistical analyses were conducted using SAS version 9.3 (SAS Institute, Cary, NC). Statistical significance was accepted as *p* of 0.05 or less. This study was approved by the Institutional Review Board at the University of North Carolina at Chapel Hill (#15-1957).

## 3. Results

A total of 6,862 stages II and III CRC patients were included in the analysis. Characteristics of the study population by age at diagnosis are shown in Table 1. Younger patients were more likely to be black or Hispanic than patients aged 50–69 and ≥70 years. The majority of younger patients were diagnosed with stage III (versus II) CRC. Tumor site varied considerably with age. In younger patients, 37% of tumors were located within the rectum and 22% in the right colon, whereas in patients over the age of 70 years, only 18% of the tumors were within the rectum, and 48% were in the right colon. A similar proportion of patients aged 50–69 years had tumors in the rectum and right colon. The proportion of tumors in the left colon was similar across all age groups.

In the analysis of the stratified subset of younger patients by race/ethnicity (Table 2), more whites had private insurance compared to both blacks and Hispanics. There were also differences by tumor site. A larger proportion of young white and Hispanic patients had rectal tumors, whereas tumors in the right colon were most common in young black patients. Although the proportion of tumors classified as high versus low grade was similar by race/ethnicity, a higher proportion of blacks had tumors with mucinous histology compared to whites and Hispanics.

Differences in county-level socioeconomic indicators by age at diagnosis are shown in Table 3. Fewer young patients lived in areas with lower median household (<$50,000) income compared to the two older groups of patients. A higher proportion of the oldest (age ≥ 70 years) patients lived in counties with lower (<10% living below poverty line) poverty, lower (<5%) unemployment rates, and higher education. There was no difference in the total number of physicians or gastroenterologists by age.

The proportion of patients who received chemotherapy differed by age at diagnosis (Table 4). Among stage II colon cancer patients, the proportion of patients who received chemotherapy decreased with increasing age. A larger proportion of stage III colon cancer patients aged <50 years and aged 50–69 years received chemotherapy than did patients aged ≥70 years. A similar pattern was observed in stages II and III rectal cancer, with the vast majority of younger patients receiving chemotherapy. The proportion of rectal cancer patients who received radiation therapy decreased with increasing age in both stages II and III. More young patients received MSI testing and had more lymph nodes (≥12) examined at surgery (Table 1).

## 4. Discussion

Our results provide a comprehensive assessment of characteristics of young-onset CRC patients across diverse settings and populations. Using a population-based sample, we found important differences in the distribution of young-onset CRC by race/ethnicity. Younger CRC patients were considerably more likely to be black or Hispanic. Moreover, these racial differences were consistent by tumor subsite, histology, and receipt of care.

There were notable racial differences in the subsite-specific distribution of young-onset CRC. Right-sided tumors predominated (39%) in younger black patients, while young white (41%) and Hispanic (33%) patients had a higher proportion of tumors located within the rectum. Young black patients also more frequently had mucinous histology, which is often associated with right-sided colon cancers. Considerable evidence suggests there are distinct CRC subtypes [30–32] and that there may be differences in these subtypes across racial groups. For example, in a recent study of* BRAF* and* KRAS* mutations among patients treated with FOLFOX-based chemotherapy in the Alliance N0147 trial [33],* KRAS* mutation was more common in black patients, while the frequency of* BRAF* mutation was the highest in tumors from whites. Other studies [34, 35] have found that, among patients with microsatellite-stable or microsatellite-low tumors, blacks have a higher frequency of* KRAS* mutations compared to whites. This difference was most pronounced in the proximal colon, with no differences in mutation frequency by race in the distal colon or rectum. Combined with the growing evidence on tumor subtypes, the differences in tumor subsite and histology we observed make a compelling argument for distinct mechanisms that drive CRC progression in racial subgroups.

We also observed suboptimal receipt of MSI testing, as well as differences in receipt by race/ethnicity. Treatment guidelines recommend that younger (age < 50) CRC patients undergo MSI testing [36], and more recently, guidelines include the option that* all* CRC patients, regardless of age, are to be considered for testing [37, 38]. Yet, less than one-third (27%) of younger patients in our study received MSI testing, and there was substantial missing data (10% missing for ages <50 years). Fewer young non-Hispanic white (25%) and Hispanic (24%) patients received appropriate testing than blacks (32%), as would be expected given the higher proportion of black patients with MSI-like histology (i.e., mucinous histology; see Table 2). Many of the studies examining the prevalence of Lynch syndrome (testing or results) have been conducted in clinic settings, where use of MSI testing ranges from 71% in comprehensive cancer centers to 15% in community hospitals [39, 40]. A different study of the Louisiana Tumor Registry [41] found that only 23% of young CRC patients received MSI testing. Suboptimal receipt of testing and considerable missing data make it difficult to draw further conclusions regarding differences in molecular subtypes of CRC by race/ethnicity. Although collection efforts have likely improved since 2010, and SEER now includes site-specific factors on MSI and* KRAS *mutation, our results highlight continued need for robust sources of molecular data at the population level.

Separately, we found that a higher proportion of younger CRC patients (both stages II and III) received “optimal” treatment, including better nodal counts from surgery, treatment at academic medical centers, enrollment in clinical trials, chemotherapy, and radiation therapy, compared to the two older groups of patients. Even in the setting of stage II colon cancer, where the absolute benefit of chemotherapy is very small, the majority of younger patients (71%) received adjuvant therapy. This may reflect physician and patient treatment preferences or advances in available therapies [21], including approval of oxaliplatin [42] and capecitabine [43] in the mid-2000s. Many of the younger stage II patients treated with chemotherapy also had high risk features, including T4 tumors (16% versus 9%), poorly differentiated histology (22% versus 19%), and inadequately sampled (<12) lymph nodes (33% versus 28%), compared to younger patients who did not receive therapy (data not shown). Despite more aggressive treatment, some research suggests that younger CRC patients have a worse prognosis than older patients of the same stage, or overall survival is similar between the two groups [4, 7, 8, 12–14, 44, 45]. A recent study [46] of colon cancer patients in the National Cancer Data Base found no difference in the relative survival of younger and older patients, even though younger patients more frequently received chemotherapy. Other pooled analyses of data from clinical trials of metastatic CRC showed that younger (age < 50 years) patients had worse progression-free and overall survival compared to patients of middle age (approximately aged 57 years), despite equivalent cancer stage and treatment [47].

An important strength of our study is the population-based sample. Data from POC studies offer a number of unique advantages for conducting population-based epidemiologic research because each participating registry area has a defined population. The age and sex distributions of patients in POC reflect those of the US population, and the SEER program includes registries with a high percentage of African Americans (Detroit, Atlanta, and Louisiana) and Hispanics (Los Angeles, Greater California, and New Mexico). POC data also provide a greater breadth and depth of information than that available solely from medical claims and/or SEER registries; detailed tumor and treatment information is abstracted from patient medical records and verified by treating physicians. This was particularly true for our assessment of receipt of chemotherapy and radiation therapy, where doctor verification substantially improved the completeness of treatment ascertainment.

The large size and diversity of this study population were also strengths that enabled us to examine CRC characteristics within population subgroups by race/ethnicity. We found that a higher proportion of Hispanic patients were diagnosed at younger ages than whites. Due to a variety of concerns, including misclassification and cultural or other differences among Hispanic and Latino groups, there has historically been limited information on cancer trends in Hispanic populations [48]. Hispanics represent the fastest-growing and youngest minority group in the US [49], and their inclusion in cancer statistics has become increasingly relevant. More recent efforts to describe cancer incidence in diverse populations show that the overall incidence rates of CRC are lower in Hispanics than in non-Hispanic whites [50], although there may be some differences in incidence by country of origin (e.g., higher incidence rates are observed in Cuban Americans) [51].

Our study population was limited to stages II and III patients. There may be different characteristics of younger CRC patients when considering early-stage or metastatic disease. For example, we observed only slight differences in county-level socioeconomic indicators among younger and older patients, but a relationship between CRC and socioeconomic status has been demonstrated most consistently in late-stage disease [52–55]. The increase in the number of younger patients diagnosed with stages II and III CRC in our study may also be a reflection of stage migration (i.e., some cases once considered stage I would now be classified as stage II); however, evidence has consistently shown meaningful increases in all stages of young-onset CRC [3]. In addition, we did not have information on genetic predisposition to CRC, either by hereditary syndrome or by a first-degree relative with a history of CRC. These data may have helped explain changes in the distribution of young-onset CRC over time, but the prevalence of hereditary syndromes in younger populations remains very low [56].

In summary, our study provides compelling evidence that characteristics of patients diagnosed with young-onset CRC differ considerably by race/ethnicity. These differences may reflect racial differences in CRC risk factors rather than disparities in diagnosis and treatment. Although exact mechanisms remain unknown, higher incidence of CRC among young black and Hispanic populations may be due to differential exposure to lifestyle-related risk factors, such as dietary patterns and a higher prevalence of obesity and sedentary behavior. Understanding differences in these risk factors by age and race/ethnicity may better elucidate reasons for the recent increase in CRC incidence in younger populations.

## Figures and Tables

**Table 1:** Characteristics of 6,862 patients diagnosed with stages II and III colorectal cancer, 1990–2010, by age at diagnosis.

	Age < 50 years (*n* = 871)		Age 50–69 years (*n* = 3,018)		Age ≥ 70 years (*n* = 2,973)	
	*n*	Weighted^1^%	*n*	Weighted^1^%	*n*	Weighted^1^%
*Demographic characteristics*						
Sex^*∗*^						
Male	472	54.0	1685	56.0	1377	44.3
Female	399	46.0	1333	44.0	1596	55.7
Race/ethnicity^*∗*^						
White, non-Hispanic	317	61.4	1490	71.5	1810	81.0
Black, non-Hispanic	200	13.3	634	11.2	449	6.7
Hispanic	189	14.7	495	9.7	386	6.7
Other	165	10.5	399	7.6	328	5.6
Insurance^*∗*^						
Private/HMO/VA/other	637	78.9	2214	76.8	1848	67.2
Medicare (only)	127	1.0	351	9.1	685	23.0
Medicaid (any)	13	14.4	254	10.6	358	9.2
None	75	5.8	128	3.6	21	0.6
*Clinicopathologic features*						
Tumor site^*∗*^						
Right colon	176	22.4	679	31.9	951	46.4
Left colon	116	15.6	332	15.1	379	15.4
Sigmoid colon	137	25.1	536	22.2	482	20.2
Rectum^2^	433	36.9	1456	30.8	1140	18.0
Stage at diagnosis^*∗*^						
Stage II	360	42.2	1437	48.7	1532	54.2
Stage III	511	57.8	1581	51.3	1441	45.8
Histologic grade^*∗*^						
Well/moderately differentiated	640	78.7	2352	82.2	2216	73.4
Poorly/undifferentiated	186	21.3	526	17.8	641	26.6
Mucinous adenocarcinoma	110	11.1	293	9.5	321	11.5
Signet ring cell carcinoma	12	0.8	28	0.7	29	1.2
MSI testing performed^3*∗*^						
Yes	68	27.3	83	9.7	44	7.1
No	140	72.7	540	90.3	423	92.9
Unknown	23		29		15	
*Treatment*						
Surgery type^*∗*^						
Local excision^4^	6	0.7	16	0.5	16	0.4
Partial colectomy or proctectomy^5^	480	56.4	1619	50.5	1466	41.1
Subtotal colectomy	240	30.7	839	38.4	1080	50.6
Total colectomy or proctectomy	98	9.1	346	6.5	249	3.8
Total proctocolectomy	38	2.4	163	3.4	139	3.5
Other	9	0.7	35	0.7	23	0.6
Lymph nodes examined^*∗*^						
0	24	1.8	89	2.0	73	1.7
1–11	258	25.8	1302	37.8	1499	45.0
≥12	560	72.4	1537	60.2	1297	53.3
*Socioeconomic indicators (census-tract-level)*						
SES index^6^						
Quintile 1	122	15.8	378	18.5	322	17.2
Quintile 2	113	21.4	350	20.3	279	20.6
Quintile 3	109	19.5	322	24.7	259	17.5
Quintile 4	113	20.8	251	16.0	276	22.2
Quintile 5	104	22.6	275	20.6	238	22.6
*Socioeconomic indicators (patient-level)*						
Hospital type^*∗*^						
Private	67	11.9	238	7.2	247	10.1
Government	182	16.1	540	17.4	387	13.5
Nonprofit	621	72.0	2212	75.4	2313	76.4
Teaching hospital^*∗*^						
No	370	45.2	1397	51.9	1564	53.9
Yes	498	54.8	1585	48.1	1374	46.1
Total bed size						
<200	194	26.1	676	25.1	710	27.2
200–400	336	39.3	1330	43.2	1335	43.6
≥400	339	34.6	982	31.6	895	29.2
Clinical trial enrollment^*∗*^						
No	728	91.7	2494	94.4	2583	98.2
Yes	67	8.3	179	5.6	74	1.8

^*∗*^ *p* < 0.05.

*Note.* The table does not include patients who did not undergo cancer-directed surgery (*n* = 167) or patients with incomplete staging information (*n* = 35); no variable had more than 10% missing data; missing observations range from 35 (tumor site) to 737 (clinical trial enrollment).

VA: Veterans Affairs; MSI: microsatellite instability; SES: socioeconomic status.

^1^Proportions weighted by sampling fraction.

^2^The term “rectal” includes both rectum and rectosigmoid junction.

^3^Microsatellite instability collected in 2010 only.

^4^Local excision includes excisional biopsy or polypectomy with or without photodynamic therapy, electrocautery, cryosurgery, laser ablation, laser excision, curette, and fulguration.

^5^Partial proctectomy includes low anterior resection and total mesorectal excision.

^6^Socioeconomic status based on composite census-tract-level indicators from Census 2000 and American Community Survey 2005–2009; limited to data collection years 2000, 2005, and 2010. Louisiana excluded due to Hurricanes Katrina and Rita's impact on populations within Gulf Coast region in 2005.

**Table 2:** Characteristics of 706 younger (age < 50 years) patients diagnosed with stages II and III colorectal cancer, 1990–2010, by race/ethnicity.

	NH white (*n* = 317)		NH black (*n* = 200)		Hispanic (*n* = 189)	
	*n*	Weighted^1^%	*n*	Weighted^1^%	*n*	Weighted^1^%
Sex^*∗*^						
Male	184	54.8	109	52.1	94	52.7
Female	133	45.2	91	47.9	95	47.3
Insurance^2*∗*^						
Private/HMO/VA/other	275	86.1	129	60.7	114	67.7
Medicaid (any)	22	10.8	48	26.1	35	19.6
None	11	3.0	16	10.5	29	9.8
Tumor site^*∗*^						
Right colon	49	19.4	62	38.5	42	26.3
Left colon	28	13.8	29	19.9	29	16.4
Sigmoid colon	44	25.9	29	17.8	33	24.3
Rectum	193	40.9	80	23.8	83	32.9
Mucinous histology^*∗*^	30	10.4	36	17.7	29	11.8
Histologic grade						
Well/moderately differentiated	246	80.7	146	81.7	140	79.2
Poorly/undifferentiated	57	19.3	39	18.3	40	20.8
Stage at diagnosis^*∗*^						
Stage II	118	42.7	86	47.4	87	38.0
Stage III	199	57.3	114	52.6	102	62.0
MSI testing performed^3^						
Yes	14	25.4	20	31.6	18	23.5
No	36	74.6	32	68.4	42	76.5
Unknown	9		1		6	
SES index^4*∗*^						
Quintile 1	11	7.7	46	37.9	45	28.0
Quintile 2	30	22.5	26	24.2	34	20.9
Quintile 3	26	16.3	18	16.3	30	27.4
Quintile 4	37	21.8	20	16.1	25	17.0
Quintile 5	43	31.7	12	5.4	8	6.7

^*∗*^ *p* < 0.05.

NH: non-Hispanic; VA: Veterans Affairs; MSI: microsatellite instability; SES: socioeconomic status.

^1^Proportions weighted by sampling fraction.

^2^Percentages do not add to 100 because some younger patients were insured through Medicare; cell sizes too small (<5) to report.

^3^MSI testing collected in 2010 only.

^4^Socioeconomic status based on composite census-tract-level indicators from Census 2000 and American Community Survey 2005–2009; limited to study years 2000, 2005, and 2010 and not including Louisiana.

**Table 3:** County-level socioeconomic indicators of 6,862 patients diagnosed with stages II and III colorectal cancer, 1990–2010, by age at diagnosis.

	Age < 50 years (*n* = 871)		Age 50–69 years (*n* = 3,018)		Age ≥ 70 years (*n* = 2,973)	
	*n*	Weighted^1^%	*n*	Weighted^1^%	*n*	Weighted^1^%
Per capita income^2*∗*^						
<$32,000	133	15.4	665	23.2	718	24.8
$32,000–50,000	575	66.0	1909	62.8	1818	58.1
>$50,000	163	18.6	444	14.0	437	17.1
Median	39637		38380		38337	
Median household income^2^						
<$50,000	176	18.8	736	24.2	691	22.8
$50,000–75,000	558	67.6	1870	63.1	1838	61.9
>$75,000	137	13.6	412	12.7	444	15.3
Median	57755		56931		57745	
% living in poverty^3*∗*^						
<10	214	32.6	619	30.0	626	36.5
10–19	409	55.5	1165	54.4	1037	52.9
≥20	99	11.9	387	15.6	299	10.7
Median	12.8		12.7		11.9	
% less than high school^*∗*^						
<10	378	37.3	1471	43.3	1614	48.7
10–19	377	45.9	1155	39.8	1042	36.4
≥20	116	16.8	392	16.9	317	14.8
Median	12.1		11.4		10.2	
Unemployment rate (%)^*∗*^						
<5	273	30.6	976	32.6	1090	36.5
5–9	437	45.7	1550	43.1	1499	45.2
≥10	161	23.7	492	24.3	384	18.3
Median	5.8		5.7		5.3	
Total physicians per 100,000						
<200	209	26.7	793	30.6	801	28.4
200–400	473	52.3	1560	48.4	1440	48.6
≥400	189	21.0	665	21.0	732	23.0
Median	268.2		268.3		268.4	
Total gastroenterologists per 100,000^3^						
<3	201	30.3	625	32.0	581	32.0
3–5	310	41.7	957	42.6	845	38.1
>5	211	28.0	589	25.5	536	29.9
Median	3.9		3.7		3.7	

^*∗*^ *p* < 0.05.

*Note.* Cutpoints based on approximate tertiles.

^1^Proportions weighted by sampling fraction.

^2^Adjusted to 2010 dollars.

^3^Poverty and total number of gastroenterologists not collected in 1990/91.

**Table 4:** Receipt of chemotherapy and radiation therapy among 6,862 patients diagnosed with stages II and III colorectal cancer, 1990–2010, by age and stage at diagnosis.

	Stage II (*n* = 3,329)			Stage III (*n* = 3,533)		
	Age < 50	Age 50–69	Age ≥ 70	Age < 50	Age 50–69	Age ≥ 70
	*n* (wt.%)	*n* (wt.%)	*n* (wt.%)	*n* (wt.%)	*n* (wt.%)	*n* (wt.%)
Received chemotherapy^1^						
Colon cancer^*∗*^						
No	71 (29.2)	465 (57.4)	809 (86.0)	43 (18.0)	156 (17.9)	439 (50.9)
Yes	121 (70.8)	281 (42.6)	131 (14.0)	180 (82.0)	621 (82.1)	390 (49.1)
Rectal cancer^*∗*^						
No	28 (17.6)	216 (34.3)	323 (52.3)	24 (6.0)	101 (9.1)	264 (42.3)
Yes	128 (82.4)	444 (65.7)	225 (47.7)	248 (94.0)	674 (90.9)	307 (57.7)
Received radiation therapy^2^						
Rectal cancer^*∗*^						
No	45 (28.5)	241 (39.0)	342 (57.1)	67 (23.3)	253 (30.1)	357 (57.8)
Yes	112 (71.5)	431 (61.0)	214 (42.9)	209 (76.7)	531 (69.9)	227 (42.2)

^*∗*^ *p* < 0.05.

*Note.* Proportions weighted by sampling fraction.

wt.: weighted.

^1^Receipt of chemotherapy includes both neoadjuvant and adjuvant (or both) chemotherapy.

^2^Receipt of radiation therapy includes postoperative and preoperative radiation.
